# Correction: Chromatin landscapes reveal developmentally encoded transcriptional states that define human glioblastoma

**DOI:** 10.1084/jem.2019019605052025C

**Published:** 2025-05-20

**Authors:** Stephen C. Mack, Irtisha Singh, Xiuxing Wang, Rachel Hirsch, Quilian Wu, Rosie Villagomez, Jean A. Bernatchez, Zhe Zhu, Ryan C. Gimple, Leo J.Y. Kim, Andrew Morton, Sisi Lai, Zhixin Qiu, Briana C. Prager, Kelsey C. Bertrand, Clarence Mah, Wenchao Zhou, Christine Lee, Gene H. Barnett, Michael A. Vogelbaum, Andrew E. Sloan, Lukas Chavez, Shideng Bao, Peter C. Scacheri, Jair L. Siqueira-Neto, Charles Y. Lin, Jeremy N. Rich

Vol. 216, No. 5 | https://doi.org/10.1084/jem.20190196 | April 4, 2019

The authors regret that, during their editing process, the data used to create the GSC 18 graph on the right in Fig. 3 e were accidentally used to create the middle GSC 18 and left GSC 1 graphs as well. Both the original and corrected Fig. 3 are shown here. This error does not affect the conclusions of the study, and the figure legend remains unchanged. The error appears in print and in PDFs downloaded before May 6, 2025.

In addition, the Fig. S2 c legend has been updated to describe the inclusion of three panels from Fig. 2; the added sentence is indicated with bold and underlined text here. The figure itself remains unchanged. The error appears in supplemental PDFs downloaded before May 14, 2025.

**Figure:**
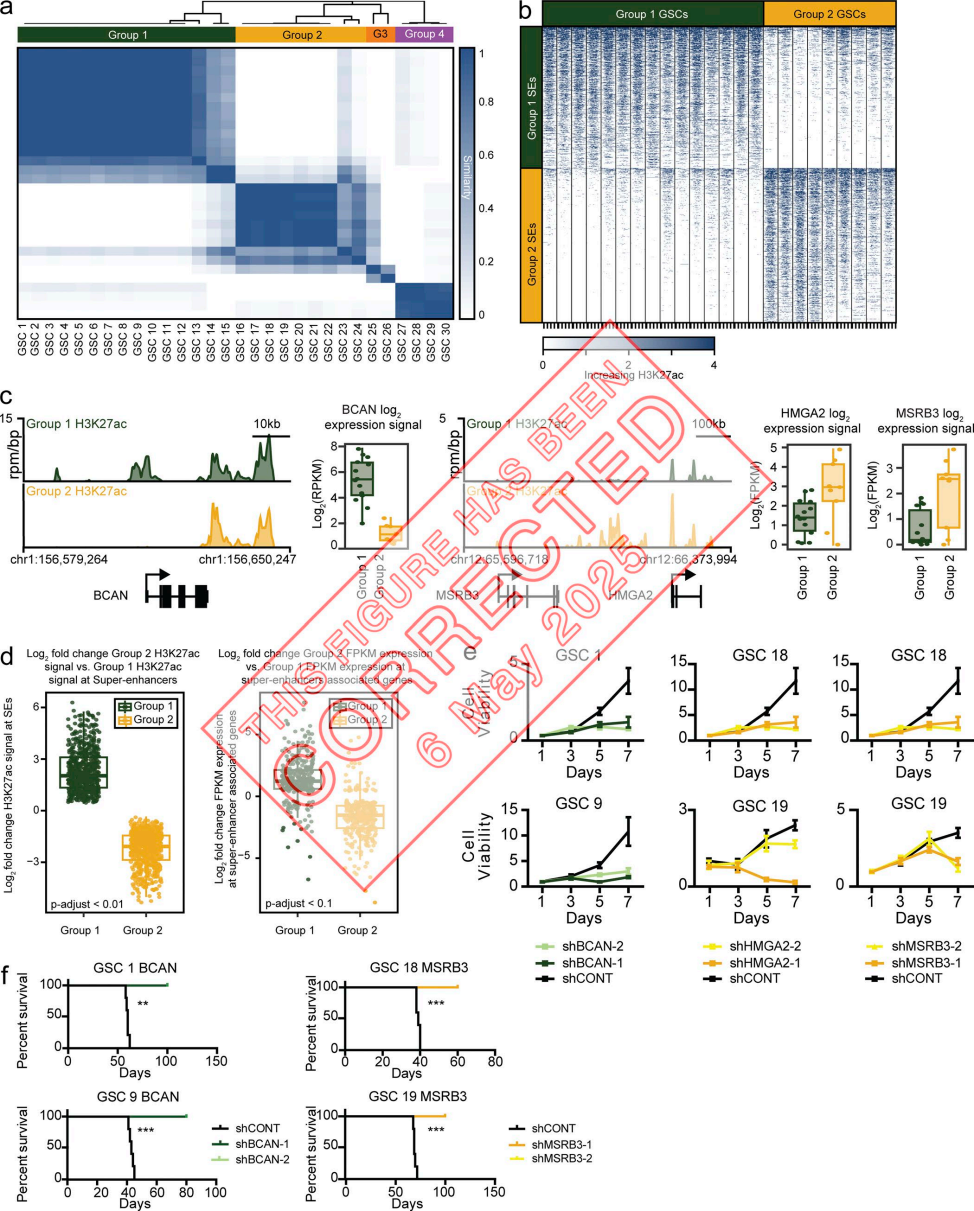


**Figure 3.**
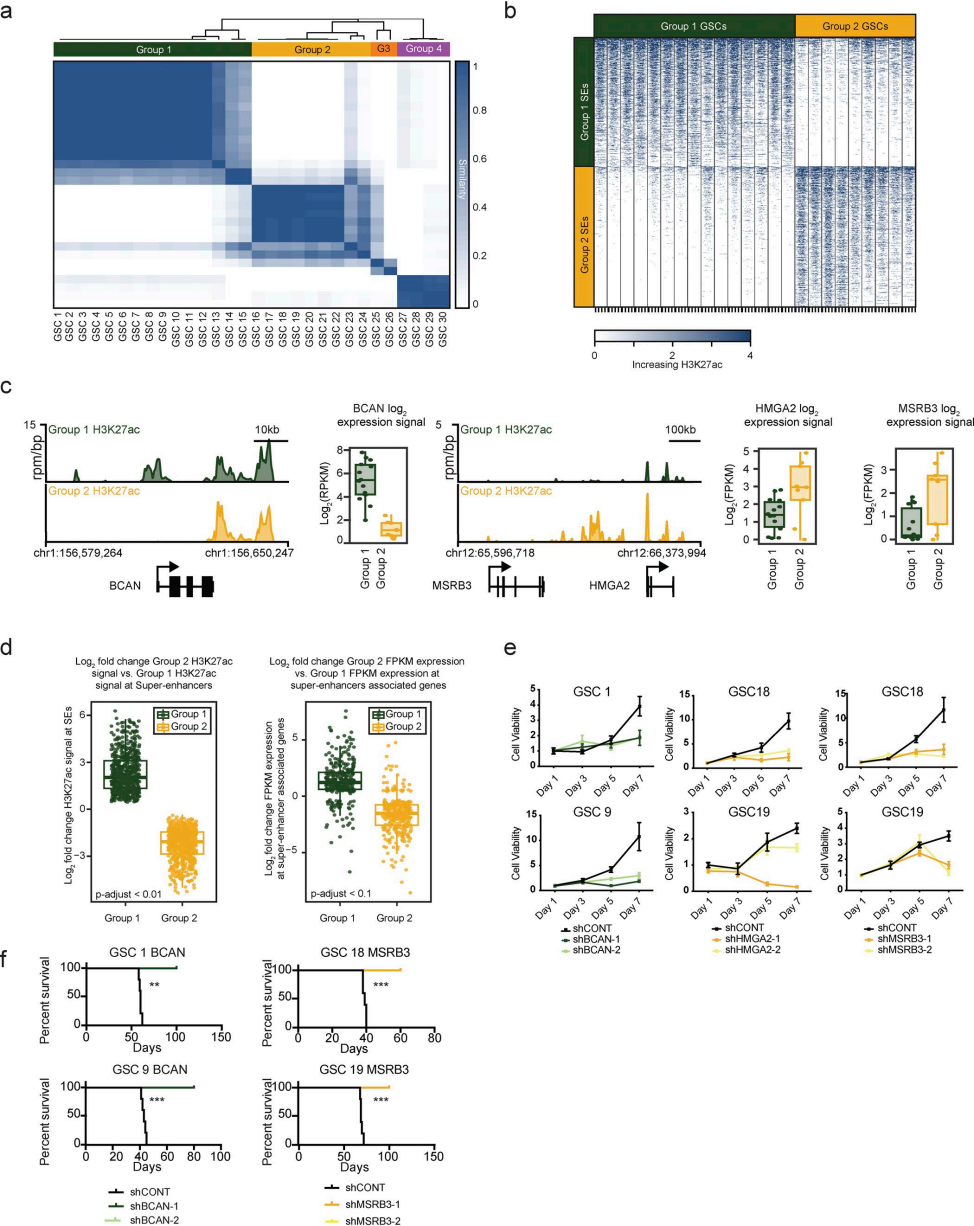
**GBMs display at least two distinct SE states. (a)** Similarity of 30 GSC samples by consensus clustering of 10% most variable regions by H3K27ac signal (n = 14,621). The GSCs clustered into four groups, group 1 GSCs (n = 15), group 2 GSCs (n = 9), group 3 GSCs (n = 2), and group 4 GSCs (n = 4). **(b)** H3K27ac occupancy around the significantly different SE (FDR-adjusted P < 0.01) between group 1 GSCs and group 2 GSCs (group 1 SEs, n = 597; group 2 SEs, n = 651). **(c)** H3K27ac activity at the BCAN, HMGA2, and MSRB3 SEs for group 1 GSCs (n = 15) and group 2 GSCs (n = 9). Gene expression from RNA-seq (FPKM, log2F- PKM) of BCAN, HMGA2, and MSRB3 shown on the right (group 1 GSCs, n = 14; group 2 GSCs, n = 9). **(d)** Change in H3K27ac activity at group 1 and group 2 SEs (FDR-adjusted P < 0.01, left) and change in expression of SE-associated genes (FDR-adjusted P < 0.1, right). **(e)** Effect of in vitro shRNA knockdown of BCAN, HMGA2, and MSRB3 on growth of group 1 and group 2 GSCs. Cell proliferation experiments were performed in biological replicates with more than four technical replicates per time point. Error bars indicated as standard deviation of four technical replicates. **(f)** Effect of in vivo shRNA knockdown of BCAN and HMGA2 on survival of mice. A log-rank test was used to calculate significance of survival differences with P values indicated as **, P ≤ 0.001 and ***, P ≤ 0.0001.

**Figure S2.**
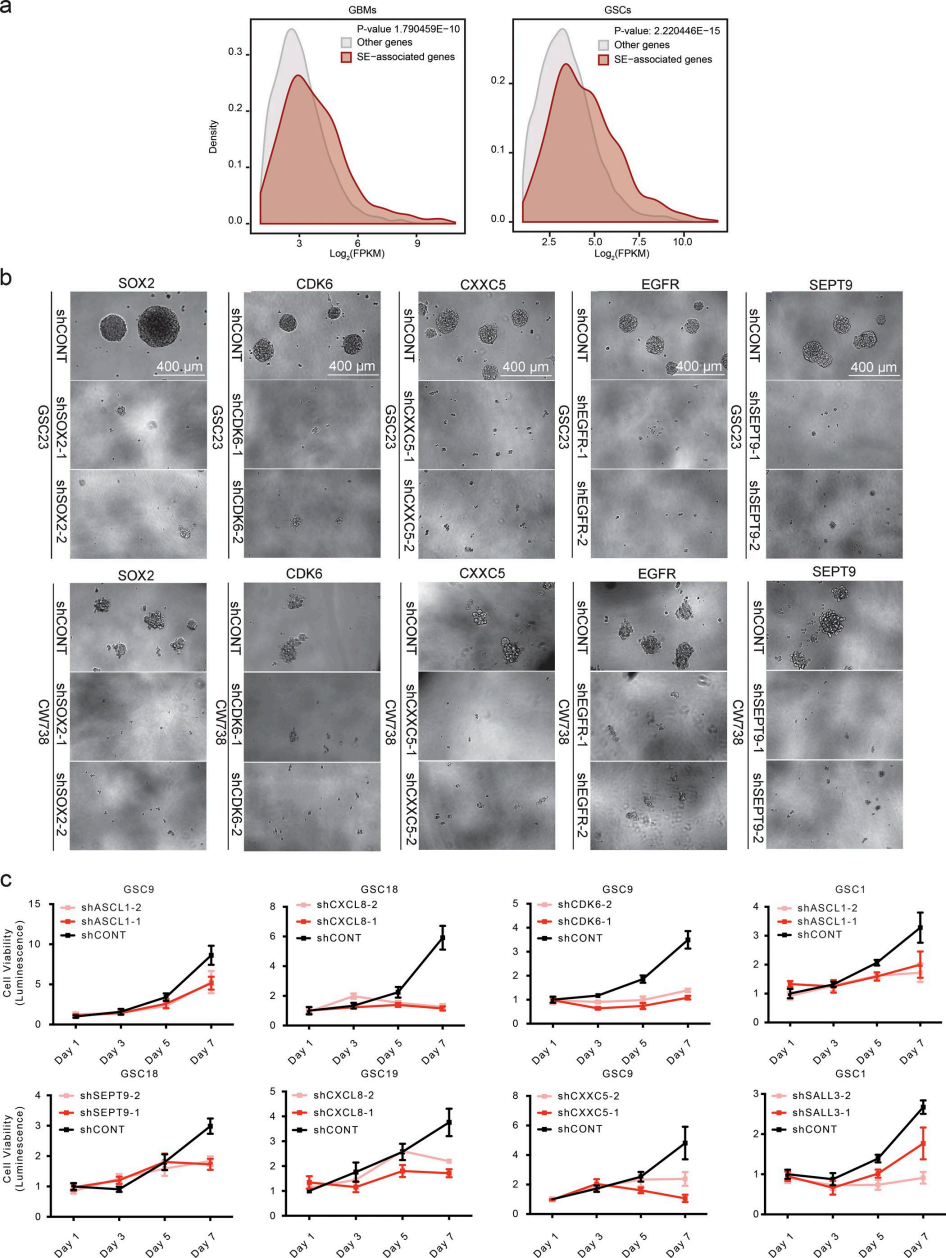
**Validation of core GSC SE genes. (a)** Distribution of expression of core SE-associated genes (n = 283) and remaining expressed genes (n = 10,968), one-sided Kolmogorov-Smirnov test (P = 1.79 ×10−10) in GBMs (left); same as on the left but for GSCs (right), one-sided Kolmogorov-Smirnov test (P = 2.22 × 10−15). **(b)** Examples of GSC neurosphere size reduction upon shRNA-mediated impairment of core stem cell identity gene expression. **(c)** Cell proliferation of GSCs following shRNA-mediated knockdown of core stem cell identity genes by using two distinct hairpins as compared with nontargeting controls. **Note that the three graphs pertaining to CDK6, SEPT9, and CXXC5 were generated from the same source data used for those graphs in Fig. 2.** Error bars indicated as standard deviation of four technical replicates.

